# Optimizing Anterior En Masse Retraction with Miniscrew Anchorage

**DOI:** 10.1155/2011/475638

**Published:** 2011-07-10

**Authors:** Pavankumar Janardan Vibhute

**Affiliations:** Department of Orthodontia, Sharad Pawar Dental College, Datta Meghe Institute of Medical Sciences (Deemed University), Room no. 101, Sawangi (Meghe), Maharashtra, Wardha 442004, India

## Abstract

In severely protrusive patients, skeletal anchorage from miniscrew is often used to avoid anchorage loss with preferred miniscrew location near centre of resistance (Cres) of posterior teeth. Biomechanical requirement for directing retraction force towards Cres of posterior teeth demands the insertion of miniscrew in loose mucosa, where risk of infection and failure increases. In addition, undesirable biomechanical side effects on anterior and posterior segments may be possible in all three planes, when continuous arch sliding mechanics are installed with miniscrew anchorage. This paper describes technique of molar-stabilizing power arm (MSPA) for simultaneous intrusion and retraction of anteriors with miniscrew placement at attached gingiva between 1st molar and 2nd premolar. Advantages of this technique include (i) the need of miniscrews placement in loose mucosa apically near the Cres of the posterior teeth is eliminated, (ii) the risk of infection and miniscrew failure is lowered since the miniscrew is placed in attached gingiva rather than the loose mucosa, and (iii) by adjusting vertical length or replacing MSPA, alteration of the retraction force vector is possible in all three planes; thus, need of removal and repositioning of the miniscrew (e.g., in correction of occlusal cant) can be eliminated.

## 1. Introduction

**Figure 1:**
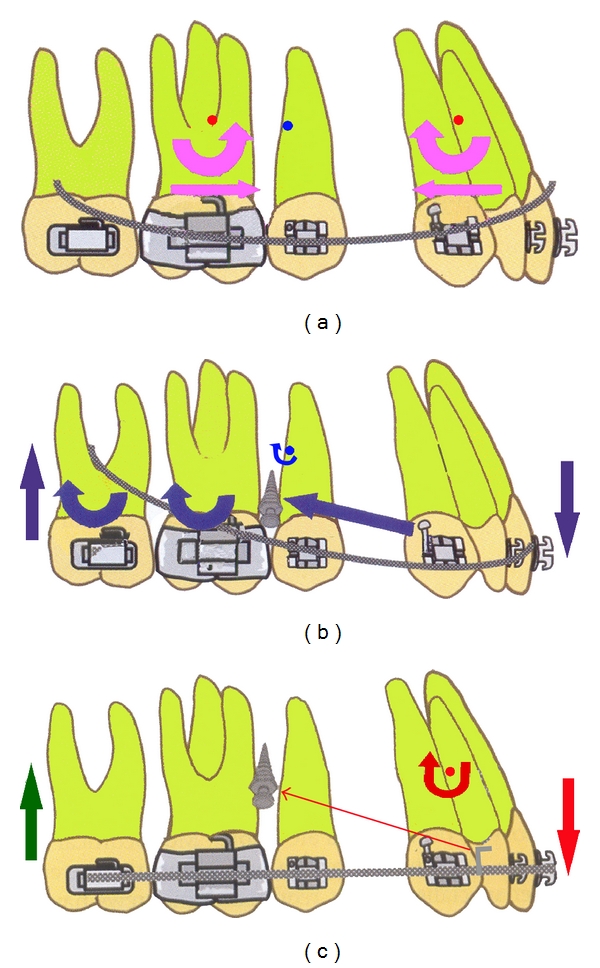
(a) Effect of space closure with conventional sliding mechanics without miniscrew. Anterior and posterior segments rotate around CR of each segment, archwire forced to bend near rotation of entire arch. These changes can easily be prevented with precurved archwires. (b) Retraction force from miniscrew anchorage with continuous archwire produces rotation of entire arch around Cres of dentition. (c) Rotation of anterior segment around Cres of anterior teeth.

Maximum anchorage is commonly required in patients with severe protrusion. Variable anchorage loss has been reported with conventional retraction by sliding mechanics in extraction cases [1–3]. Use of miniscrew for reinforcement of orthodontic anchorage has become increasingly popular in recent years, especially for the space closure in maximum anchorage cases [4]. Miniscrews are convenient, save time, and do not require patient cooperation [5–8]. Sliding mechanics are most commonly followed for space closure with miniscrew [9]. For achieving the direction of force vector towards the centre of resistance (Cres) of posterior teeth with retraction and intrusion of anterior teeth, position of miniscrew is preferred in apical portion, between 2nd premolar and 1st molar or 1st and 2nd molars, near Cres of posterior segment [10]. This biomechanical requirement and limited corridor of attached gingiva demand the insertion of miniscrew to be in loose mucosa, where risk of infection and failure increases [11–16]. The sliding mechanic retraction assembly with direct anchorage from miniscrew reported biomechanical drawbacks. These biomechanical side effects may be in three planes and inherent with the use of continuous arch sliding mechanics with miniscrew [17]. With conventional sliding mechanics without skeletal anchorage, extraction spaces are typically closed by attaching retraction assembly between an anterior archwire hook and second molars. In the Sagittal plane, the anterior and posterior segments rotate around their respective centre of rotation (CR), which causes bowing of archwire (Figure 1(a)). Use of precurved archwire can prevent this. 

**Figure 2:**
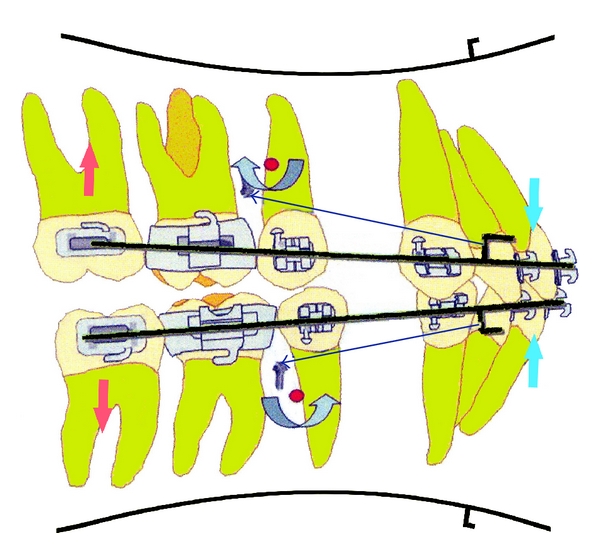
Intrusive force on posterior teeth causing posterior open bite and anterior deep bite.

Incorporation of miniscrew for anchorage reinforcement produces different mechanics. Because the force used during retraction is not reciprocal, posteriorly it is negated not by teeth but by miniscrew. As a result, either the entire arch (Figure 1(b)) or the anterior segment (Figure 1(c)) rotates around the CR. In cases of severe protrusion, where maximum anchorage is required in both arches, these mechanics produce posterior open bite and anterior deep overbite (Figure 2). The use of precurved archwire results in an even stronger intrusive force on posterior segment. Therefore, these mechanics have to be used cautiously in low-angle and deep-bite cases. Symptoms of temporomandibular disorders (TMD) may develop because of bilateral loss of contact in posterior stops. To avoid this problem clinician must check the posterior occlusion in centric relation, making sure that some posterior contact exists bilaterally. Therefore, this situation demands the placement of additional anterior miniscrew for intrusion.

In the horizontal plane, morphology of upper molars provides less resistance to rotation; upper molars tend to tip more than the lower molars. Maxillary molars tend to tip palatally more than mandibular molars, which lead to development of buccal cross-bite.

This paper shows how the biomechanics of anterior retraction are balanced in all three planes with stabilizing molars and eliminating the need of miniscrew placement in loose mucosa or additional miniscrews in anterior region.

## 2. Technique

**Figure 3:**
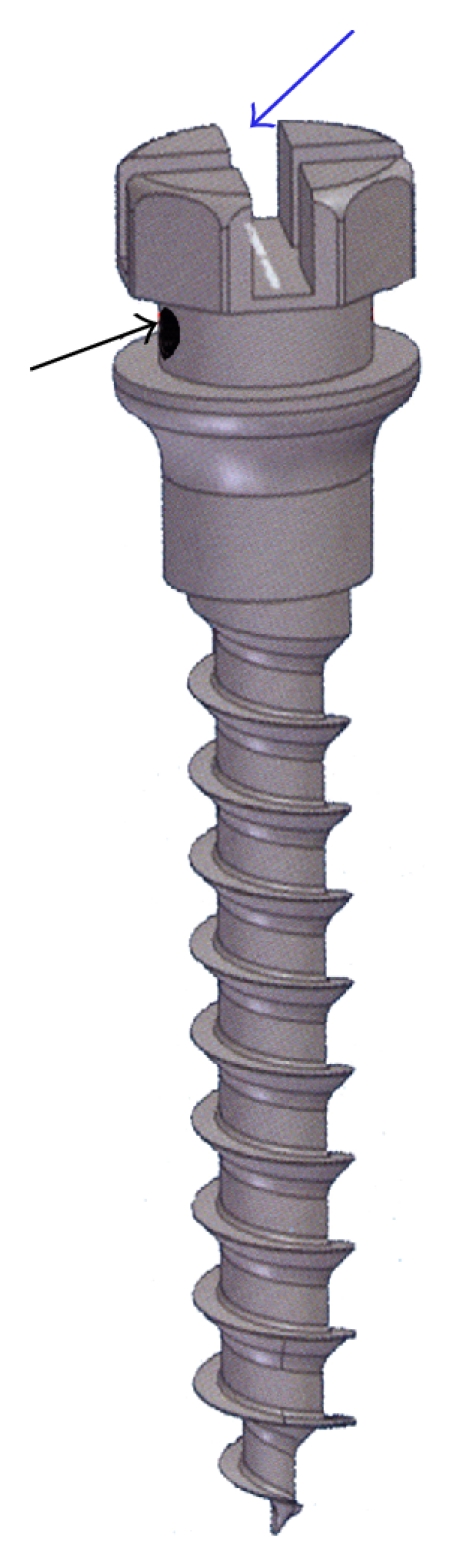
Dual-head miniscrew having rectangular slot and ligature hole. Hole used for threading ligature wire and securing MSPA in slot of bracketed head miniscrew.

**Figure 4:**
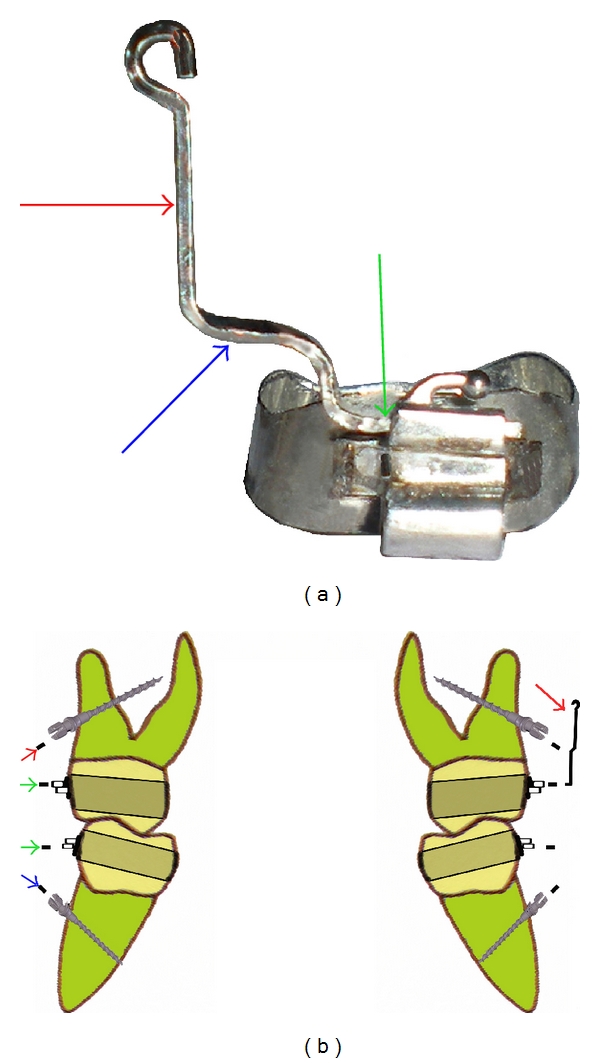
(a) MSPA consists of three parts: vertical-hooked arm (red arrow), horizontal middle part (blue arrow), and distal straight end section inserted in auxiliary molar tube (green arrow). (b) Place 1st- and 3rd-order bends as required in middle horizontal section of MSPA so that it passively engages the slot of miniscrew with insertion of distal end section into auxiliary molar tube.

**Figure 5:**
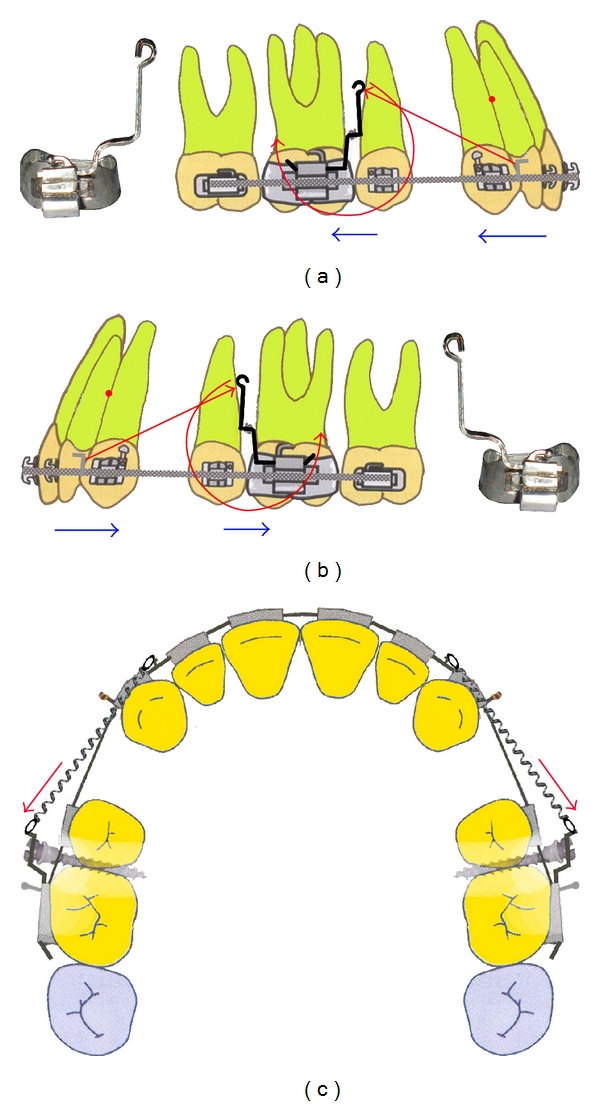
(a, b) Right and left lateral views of biomechanics of space closure with MSPA. (c) Transverse view of miniscrew biomechanics.

(1) Insert miniscrews as needed for anchorage between 1st molar and 2nd premolar roots in attached gingiva region. Use miniscrew with dual top head (bracket head type) having rectangular slot and a ligature hole beneath it (Figure 3). Recommended angle of the implant insertion to long axis of the teeth have ranged from 10°–20° in mandible and from 30°–40° in maxilla. Slot in the head of the miniscrew placed preferably parallel to occlusal plane which helps in stabilizing and functioning of MSPA.

(2) Construct Molar Stabilizing Power Arm (MSPA) in 0.017′′ × 0.025′′ stainless steel (SS) (for 0.018′′ appliance) or 0.019′′ × 0.025′′ SS (for 0.022′′ appliance). It has three parts: vertical-hooked-arm, middle part to be engaged in miniscrew head slot and horizontal distal end section for insertion into auxiliary molar tube (Figure 4(a)). Determine the length of MSPA's vertical-hooked-arm in accordance the depth of buccal vestibule, and angle this arm to position the hook near the Cres of posterior segments bilaterally. Bend the hooks into rounded shapes to avoid mucosal impingement.

 (3) Since angles of the implant insertion to long axis of the teeth have ranged from 10°–40°, plane and distance of “slot depth” of miniscrew head may not be necessarily parallel to plane of auxiliary molar tube. Place 1st and 3rd order bends as required in middle horizontal section of MSPA, so that it passively engages the slot of miniscrew after insertion of distal end section into auxiliary molar tube (Figure 4(b)).

(4) Thread a ligature wire through the hole beneath slot and secure the power arm to miniscrew head by twisting the ligature wire and then tuck in the wire ends. If bracketed head miniscrew without ligature hole is used, then power arm may be secured with ligature tie same way as followed in bracket. 

(5) Connect a nickel titanium coil spring from the hook of the MSPA to anterior archwire hook (3–5 mm long). In maxilla, coil spring will generate upward and backward retraction forces (Figure 5); additionally, posterior teeth receive distalizing forces. 

(6) Adjust the hooked vertical-hooked arm of the MSPA, so that the retraction assembly clears the alveolar mucosa.

**Case**

**Diagnosis**

**Figure 6:**
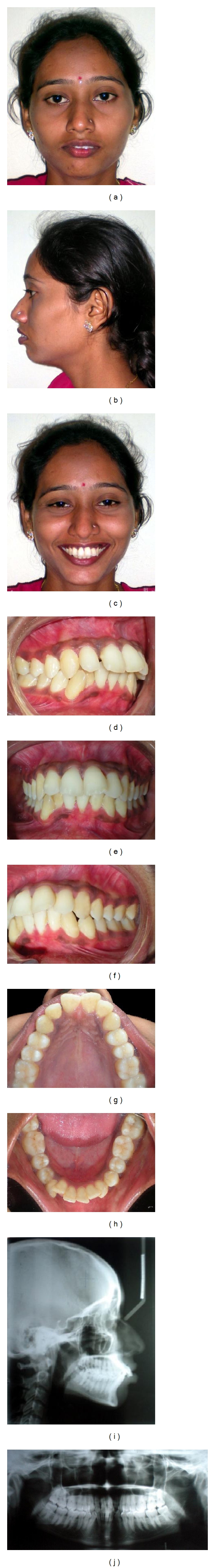
(a–j) Pretreatment photographs and radiographs.

A 26-year-old female patient presented with chief complaint of bimaxillary protrusion, convex profile with incompetence, and protrusive lips (Figure 6). After clinical and cephalometric examinations, she was having diagnosed as skeletal mild Class II and dental class I malocclusion with severe bialveolar protrusion, crowding, and average growth pattern.

**Treatment Plan**

Treatment plan called for orthodontic treatment with all 1st premolar extractions, to resolve proclination and crowding considered as maximum anchorage case. It was planned to retract canines initially and shortly to allow the de-crowding of incisors for alignment and consolidation of anterior segment, before proceeding to en masse retraction. For initial canine retraction, and later for en-masse retraction, use of miniscrew was decided for the anchorage with sliding mechanics. Width of attached gingiva in maxilla was less than mandible in molar region. With maxilla, it was planned to insert the miniscrew more occlusally in attached gingiva than in mandible. Location of mucogingival junction was quite apically and satisfactory as site for miniscrew insertion. Due to considerable biomechanical side effects of the conventional direct pull from miniscrew, it was decided to use MSPA and to have retraction force from the MSPA in maxilla and from the miniscrew head in mandible, which will be connected to auxiliary molar tube.

**Treatment Progress**

**Figure 7:**
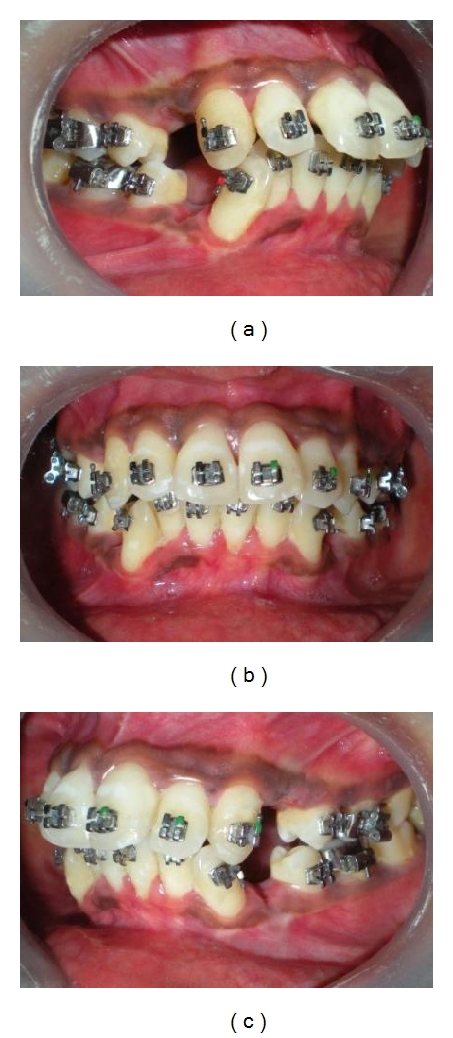
(a, b, c) 0.022′′ PEA appliance strapped up, all four 1st premolars extracted.

**Figure 8:**
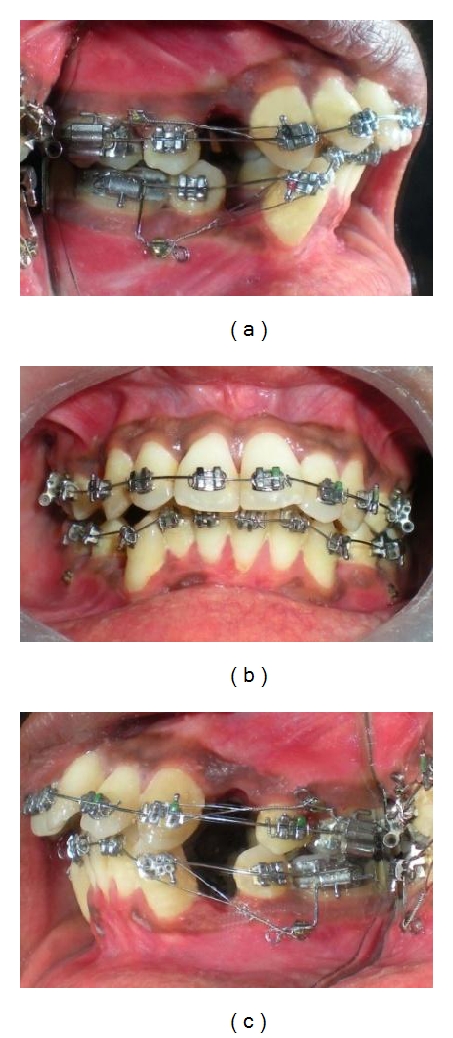
(a, b, c) Miniscrews were placed between roots of 1st molar and 2nd premolar at keratinized gingiva in maxilla and at mucogingival junction in mandible; canine retractions were done only initially and shortly to decrowd the incisors.

0.022′′ slot preadjusted edgewise appliance (PEA) brackets were bonded, and bands were placed on 1st molars. All four first premolars were extracted (Figure 7). In maxilla, miniscrews [18] (1.5 mm diameter, 11.6 mm long, bracket head type (Aarhus Mini-Implant 2920 Charlottenlund ScanOrto Denmark Hans Edvard Teglers Vej 2)) were placed between roots of 1st molar and 2nd premolar at keratinized gingiva. Corridor of keratinized gingiva was sufficient in mandible so miniscrews (Aarhus Mini-Implant 2920 Charlottenlund ScanOrto Denmark Hans Edvard Teglers Vej 2) were inserted at mucogingival junction. During alignment and leveling, MSPAs (0.019′′ × 0.025′′ SS) without vertical-hooked arm were inserted into auxiliary molar tubes in both arches (Figure 8). Initial canine retractions were carried with immediate loading [19], and retraction forces with active lacebacks were applied from the miniscrew, which were connected to auxiliary molar tubes.

Alignment and consolidation of maxillary anterior teeth were completed earlier than mandibular, within three months. Due to more coronal location of miniscrew in maxilla and need of intrusive component during retraction, old MSPAs (0.019′′ × 0.025′′ SS) were replaced with new ones, having vertical-hooked-arm. En-masse retraction was started earlier in maxillary arch due to earlier alignment and consolidation of anterior segment and excessive overjet. Space closure was started with closed coil spring by sliding mechanics considered as a case of maximum anchorage.

Due to considerable apical distance of mandibular miniscrew head from auxiliary molar tube, MSPAs without vertical-hooked arm were used in mandible and retraction force for initial canine retraction and later for space closure was delivered directly from miniscrew head, which were connected to auxiliary molar tube.

**Figure 9:**
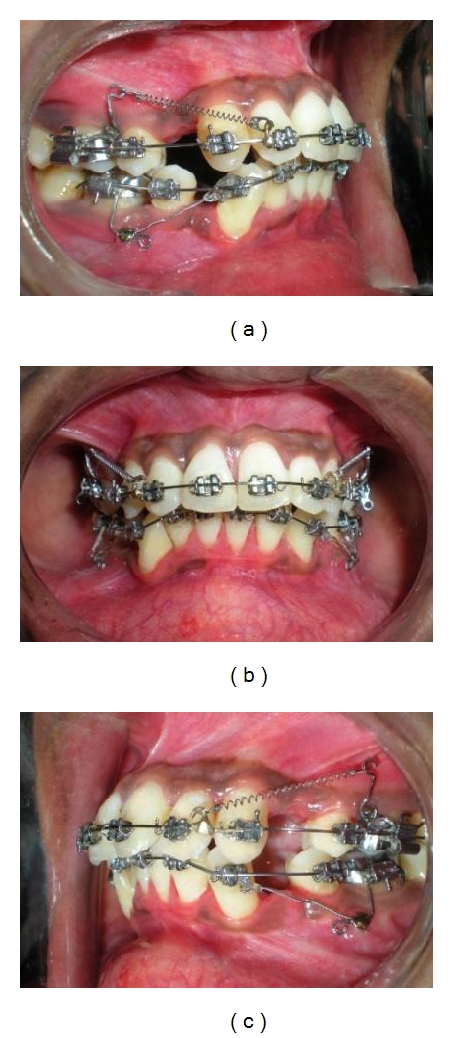
(a, b, c) MSPAs were inserted in the auxiliary molar tube and slot of the miniscrew head at all four quadrants. Mandibular MSPAs were without vertical-hooked-arm. Due to less initial crowding and increased overjet, maxillary en-masse retraction was started earlier. Mandibular canine retraction continued further keeping them in class I relation.

**Figure 10:**
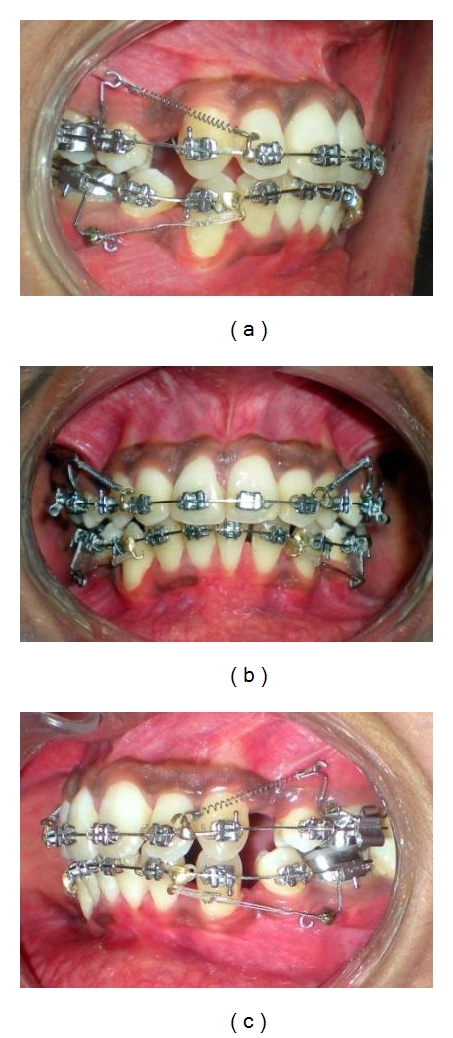
(a, b, c) Simultaneous en-masse retraction was continued in both arches. Closed coil springs were used for the en-masse retraction which were stretched and engaged posteriorly over MSPA and anteriorly to hook soldered on archwire. Although miniscrews were placed towards occlusal level, forces exerted by the springs were directed apically and towards Cres of posterior teeth.

Precurved and coordinated archforms of 0.018′′ × 0.025′′ SS continuous archwires were used in both arches to prevent the bite from deepening during retraction as per conventional sliding mechanics with PEA. Sentalloy (registered trademark of GAC Inc., 355 Knickerbocker Avenue, International, Bohemia, NY 11716 USA; http://www.gacintl.com,) NITI Closed coil springs, each exerting a retraction force of 250 gm–300 gm, were engaged between MSPAs and soldered anterior hooks on the archwire (Figures 9 and 10). No class II or class III interarch elastics were used throughout treatment except for the anterior diagonal elastics, just before completion of space closure for midline correction.

**Figure 11:**
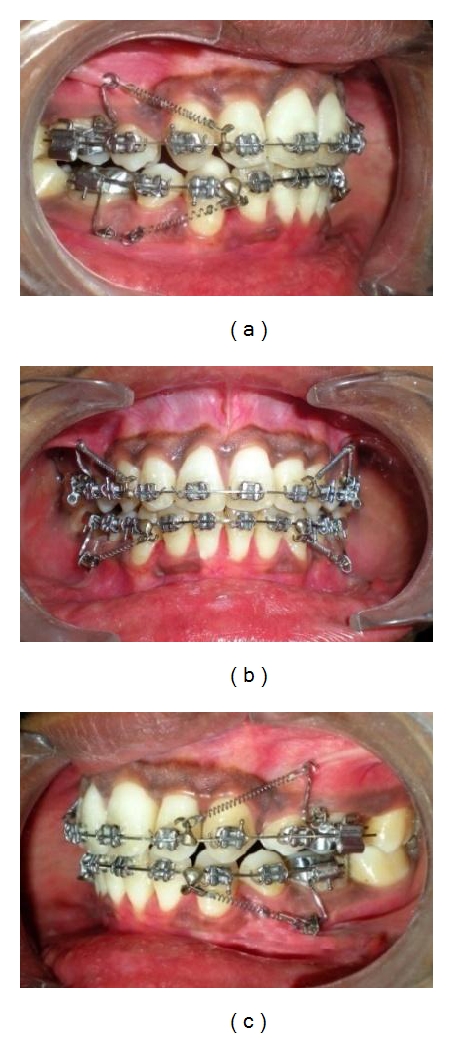
(a, b, c) Midlines were corrected just before completion of space closure using interarch anterior diagonal elastics.

**Figure 12:**
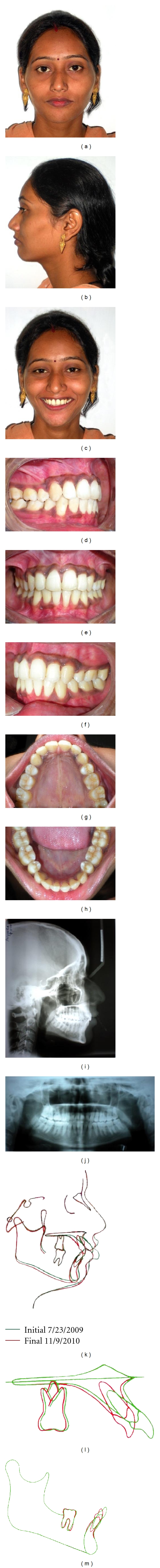
(a–j) Posttreatment photographs and radiographs. (k) Overall pre- and posttreatment superimposition depicts profile improvements. (l, m) Maxilla and mandible pre- and posttreatment superimpositions.

At the end of 13 months, space closure in both arches was completed without adverse effects, that is, posterior open bite and deepening of bite. Bimaxillary proclination was resolved without molar intrusion and anchorage loss (Figures 11 and 12). MSPA and retraction assembly did not show distortion or any signs of soft-tissue irritation. Throughout treatment, none of the miniscrews had shown any signs of loosening or its failure. MSPA and miniscrews were removed before the stage of finishing and settling of occlusion.

**Treatment Results**

After 15 months of total active treatment, goals had been achieved. Patient showed good class I dental relationship with upper and lower anterior teeth retracted and uprighted into near normal position over basal bone (Table 1). With retraction of upper and lower lips, facial profile and smile were improved dramatically. Upper and lower wraparound retainers were delivered.

## 3. Discussion

In the case shown here, MSPA provided reliable approach of skeletal anchorage. MSPA worked in three ways. (i) It stabilized the molar in three planes; intrusive forces on molar due to sliding mechanics are counterbalanced. Additionally, MSPA eliminated the constrictive effect on molars. Thus, need of bonding second molar and placement of transpalatal arch has been eliminated. (ii) Miniscrews although were positioned in attached gingival, MSPA provided the point of attachment near Cres of posterior segment for retraction assembly. (iii) While placing the miniscrew in keratinized gingiva, it provided posterior and superior vector of force, which was required for intrusion in anterior teeth. In spite of using precurved archwires and miniscrew, creation of posterior open bite and anterior deep bite has avoided efficiently.

In mandibular arch, direction of retraction force was satisfactory and resulted in remarkable amount of curve of spee correction. Stabilization of lower molar has been performed well with a part of MSPA. FMA opened by 1° suggested the distal thrust on the all 1st molars and had wedging effect posteriorly, causing minor clockwise rotation of mandible. Taking into consideration the patient's age and fully erupted 2nd and 3rd molars, major distal movement of 1st molars may not have occurred, but expanded the scope for this sound biomechanical design.

Inserting torques in the miniscrew was in clockwise direction. MSPA was favorable for the mechanics on the right side of maxillary arch, which tightens the screw. But one can avail the miniscrews with reverse threads (left-handed thread type) on left side for similar biomechanics so that if de-torquing rotational force was exerted by the MSPA, it augments its firmness [20].

This technique shows numerous advantages.

1. Force system has balanced in such a way that posterior intrusive forces on molars have been balanced by miniscrew.
2. With long vertical-hooked-arm, need of placement of miniscrew in apical region, near to Cres of posterior teeth was eliminated.
3. Need of miniscrew placement in loose mucosa may be eliminated, since risk of infection and failure is more.
4. Direction of force vector in transverse, horizontal, and vertical planes may be adjusted without changing the position of miniscrew, but only by either adjusting or replacing the power arm. Canted occlusal planes and shifted midlines can be corrected by only adjusting the length of power arm without changing miniscrew position.
5. Conventionally, once miniscrew placed, designing of biomechanics is dictated by the position of miniscrew, but here, with adjustable and replaceable power arm, clinician can dictate the biomechanics throughout treatment with miniscrew in same location.
6. Intermaxillary elastics between the posterior teeth are no longer required, as risk of developing posterior open bite is reduced.
7. Vertical-hooked-arm of MSPA may be adjusted in buccopalatal direction. Therefore, curvature in the arch form does not cause impingement of retraction spring over alveolar mucosa.
8. Since posterior teeth are stabilized, rotational effect on occlusal plane is reduced and thus helps in eliminating the chance of developing anterior deep bite.

## Figures and Tables

**Table 1:** Cephalometric analysis data.

Parameter	Pretreatment	Posttreatment
SNA	80.7°	79.3°
SNB	75.4°	74.5°
ANB	5.3°	4.7°
FMA	23°	24°
U 1 to NA degree	35.8°	18.5°
L 1 to NB degree	31.9°	23.9°
U 1 to NA (mm)	10 mm	4 mm
L 1 to NB (mm)	9 mm	2 mm
Interincisal angle	107°	132.9°
PFH/AFH	73%	73%
FH/OP degree	4°	11°
Max 1-SN	166.5°	97.8°
IMPA	105°	97°
Z angle	42°	63°
Upper lip to E line	−1 mm	−2 mm
Lower lip to E line	6 mm	0 mm
U 1 to A-Pog (mm)	13 mm	5 mm
Holdaway ratio	6	3
Wits	6 mm	1 mm

## References

[1] Kim TK, Kim JT, Mah J, Yang WS, Baek SH (2005). First or second premolar extraction effects on facial vertical dimension. *Angle Orthodontist*.

[2] Ong HB, Woods MG (2001). An occlusal and cephalometric analysis of maxillary first and second premolar extraction effects. *Angle Orthodontist*.

[3] Kocadereli I (1999). The effect of first premolar extraction on vertical dimension. *American Journal of Orthodontics*.

[4] Park HS, Bae SM, Kyung HM, Sung JH (2001). Micro-implant anchorage for treatment of skeletal Class I bialveolar protrusion. *Journal of Clinical Orthodontics*.

[5] Costa A, Raffini M, Melsen B (1999). Microscrews as orthodontic anchorage. *International Journal of Adult Orthodontics & Orthognathic Surgery*.

[6] Bae SM, Park HS, Kyung HM, Kwon OW, Sung JH (2002). Clinical application of micro-implant anchorage. *Journal of Clinical Orthodontics*.

[7] Carano A, Velo S, Leone P, Siciliani G (2005). Clinical applications of the Miniscrew Anchorage System. *Journal of Clinical Orthodontics*.

[8] Papadopoulos MA, Tarawneh F (2007). The use of miniscrew implants for temporary skeletal anchorage in orthodontics: a comprehensive review. *Oral Surgery, Oral Medicine, Oral Pathology, Oral Radiology and Endodontology*.

[9] Park HS (2000). A new protocol of the sliding mechanics with micro-implant anchorage (MIA). *Korean Journal of Orthodontics*.

[10] Park HS, Kwon OW, Sung JH (2005). Microscrew implant anchorage sliding mechanics. *World Journal of Orthodontics*.

[11] Park HS, Jeong SH, Kwon OW (2006). Factors affecting the clinical success of screw implants used as orthodontic anchorage. *American Journal of Orthodontics and Dentofacial Orthopedics*.

[12] Kravitz ND, Kusnoto B (2007). Risks and complications of orthodontic miniscrews. *American Journal of Orthodontics and Dentofacial Orthopedics*.

[13] Park HS (2003). Clinical study on success rate of microscrew implant for orthodontic anchorage. *Korean Journal of Orthodontics*.

[14] Cheng SJ, Tseng IY, Lee JJ, Kok SH (2004). A prospective study of the risk factors associated with failure of mini-implants used for orthodontic anchorage. *International Journal of Oral and Maxillofacial Implants*.

[15] Cha BK, Lee YH, Lee NK, Choi DS, Baek SH (2008). Soft tissue thickness for placement of an orthodontic miniscrew using an ultrasonic device. *Angle Orthodontist*.

[16] Wilmes B, Rademacher C, Olthoff G, Drescher D (2006). Parameters affecting primary stability of orthodontic miniimplants. *Journal of Orofacial Orthopedics*.

[17] Jung MH, Kim TW (2008). Biomechanical considerations in treatment with miniscrew anchorage. Part 1: the sagittal plane. *Journal of Clinical Orthodontics*.

[18] Melsen B, Verna C (2005). Miniscrew implants: the aarhus anchorage system. *Seminars in Orthodontics*.

[19] Melsen B, Costa A (2000). Immediate loading of implants used for orthodontic anchorage. *Clinical Orthodontics and Research*.

[20] Motoyoshi M, Hirabayashi M, Uemura M, Shimizu N (2006). Recommended placement torque when tightening an orthodontic mini-implant. *Clinical Oral Implants Research*.

